# Liver Accumulation of *Plasmodium chabaudi*-Infected Red Blood Cells and Modulation of Regulatory T Cell and Dendritic Cell Responses

**DOI:** 10.1371/journal.pone.0081409

**Published:** 2013-11-27

**Authors:** Márcia M. Medeiros, Henrique B. da Silva, Aramys S. Reis, Renato Barboza, Joanne Thompson, Maria Regina D'Império Lima, Cláudio R. F. Marinho, Carlos E. Tadokoro

**Affiliations:** 1 Instituto Gulbenkian de Ciência, Oeiras, Portugal; 2 Department of Immunology, Instituto de Ciências Biomédicas, Universidade de São Paulo, São Paulo, Brazil; 3 Department of Parasitology, Instituto de Ciências Biomédicas, Universidade de São Paulo, São Paulo, Brazil; 4 Institute of Immunology and Infection Research, School of Biological Sciences, University of Edinburgh, Edinburgh, United Kingdom; Federal University of São Paulo, Brazil

## Abstract

It is postulated that accumulation of malaria-infected Red Blood Cells (iRBCs) in the liver could be a parasitic escape mechanism against full destruction by the host immune system. Therefore, we evaluated the *in vivo* mechanism of this accumulation and its potential immunological consequences. A massive liver accumulation of *P. c. chabaudi* AS-iRBCs (Pc-iRBCs) was observed by intravital microscopy along with an over expression of ICAM-1 on day 7 of the infection, as measured by qRT-PCR. Phenotypic changes were also observed in regulatory T cells (Tregs) and dendritic cells (DCs) that were isolated from infected livers, which indicate a functional role for Tregs in the regulation of the liver inflammatory immune response. In fact, the suppressive function of liver-Tregs was *in vitro* tested, which demonstrated the capacity of these cells to suppress naive T cell activation to the same extent as that observed for spleen-Tregs. On the other hand, it is already known that CD4+ T cells isolated from spleens of protozoan parasite-infected mice are refractory to proliferate *in vivo*. In our experiments, we observed a similar lack of *in vitro* proliferative capacity in liver CD4+ T cells that were isolated on day 7 of infection. It is also known that nitric oxide and IL-10 are partially involved in acute phase immunosuppression; we found high expression levels of IL-10 and iNOS mRNA in day 7-infected livers, which indicates a possible role for these molecules in the observed immune suppression. Taken together, these results indicate that malaria parasite accumulation within the liver could be an escape mechanism to avoid sterile immunity sponsored by a tolerogenic environment.

## Introduction

In humans, the lack of infected Red Blood Cells (iRBCs) that carry asexual mature forms of *Plasmodium falciparum* from the peripheral circulation occurs through adherence of these cells to the micro vascular endothelium in various organs [1], [2]. It is thought that iRBC accumulation is a parasitic escape mechanism to avoid passage and destruction inside the spleen [3], a possibility which presents important implications for the development of severe malarial disease. However, new insights have also demonstrated the importance of accumulation to parasite growth [4].

One of the main parasite molecules linked to cytoadhesion of iRBCs to endothelial cells is *P. falciparum* erythrocyte membrane protein-1 (PfEMP-1) [5]–[8]. The endothelial ligands for this molecule include CD36, thrombospondin, ICAM-1, PECAM-1, VCAM-1, chondroitin-4-sulphate, ELAM-1, and P-selectin [9]–[16].

Animal models for human infections are needed for improved quantification and qualification of the pathological events that occur during infections. In mice, accumulation of iRBCs that contain mature parasitic forms has been observed in *Plasmodium chabaudi (P. c.) chabaudi* AS-infected mice [17], [18]. Antigenic variations of *cir* multigenic family and accumulation have also been observed in a *P. c. chabaudi* infection model [19], which suggests that proteins expressed by *cir* genes are involved in this phenomenon [18], as was proposed for PfEMP-1. Other studies have demonstrated that *P. c*-iRBCs adhere to CD36 *in vitro* in a γ-interferon-dependent manner and *in vivo* at tissues of multiple organs [20]. Despite the differences between molecules that affect cytoadhesion of iRBCs in animal models or human infections, the results obtained by using *P. c. chadaudi* could be relevant to understand the effect of iRBCs cytoadhesion inside organs in both species.

Accumulation of *P. c. chabaudi* AS mature forms inside the liver could not only be a relevant malarial parasite escape plan against further destruction inside the spleen, but could also be a survival strategy through the suppression of the immune response against malarial antigens. As documented by studies with LPS stimulation or chronic viral infections [21]–[23], the liver can be considered an immune tolerogenic organ due to the presence of sub-optimal antigen presentation conditions, such as down regulation of MHCII, CD80, and CD86 expression, and release of anti-inflammatory molecules such as IL-10 and PGE2 by liver sinusoidal endothelial cells (LSECs) and Kupffer cells (KFCs). This immune system suppression could also promote host survival because the failure to control immunopathology that results from excessive inflammation is an important factor in the development of severe malaria [24]. Regulatory T cells (Tregs) are key mediators in the maintenance of tolerance against self and non-self antigens [25], [26], as well as the restraint of excessive immunological responses [27]. Similar to those of different parasite strains in human and mouse hosts, the nature and outcome of malarial infections also seem to be governed by the balance between pro-inflammatory and anti-inflammatory immune responses, and some studies point to the participation of Tregs in this immunological balance [28]–[30]. Indeed, a small number of Tregs was reported to contribute to immunopathology during the chronic infection phase [31].

A wide range of results was reported from several initial Treg depletion trials, using monoclonal antibodies (mAbs) against CD25 that were carried out by different researchers. In particular, it was shown that CD25+ Treg depletion was incomplete after treatment, which was accompanied by a fast recovery of the Treg population [32], higher parasitemia in CD25-depleted animals compared with control animals [33], lower parasitemia levels after CD25-depletion [34], [35], and reduced death rates from cerebral malaria (CM) in *P. berghei* ANKA-infected mice [36]. However, the use of anti-CD25 mAbs also removes activated CD25+ effector T cells (Teffs) and compromises the interpretation of these results. More recently, it was demonstrated that depletion of Tregs did not alter the parasitemia levels or lethality in *P. berghei* ANKA-infected animals that expressed diphtheria toxin (DT) receptor on Foxp3+ cells (hence Tregs were depleted after DT injection). Nonetheless, the increased Treg numbers after *in vivo* treatment with IL-2 could protect animals against CM and decrease the parasite load throughout the body [37]. From these studies, one possible hypothesis is that the rapid increase in Treg numbers may control expansion of T cells and further sequestration of T cells inside the brain tissue, avoiding the complications seen in CM cases. An increase in Treg numbers was observed in animals after *P. berghei* ANKA infection, although these Tregs did not migrate to the CNS [38]. Therefore, an increased accumulation of Tregs during infections, even in organs such as the spleen and liver, might ultimately protect against the development of severe malarial pathology and host death. Additionally, it may be postulated that Tregs contribute to control the immune system activation within the liver, thus retaining it as a temporary parasite reservoir.

Dendritic cells (DCs) also have important roles within the murine immune system during experimental malaria infections. It has been shown that DCs are very important in the presentation of *P. yoelii* antigens to CD8+ T cells [39]–[41], leading to IFN-γ production that is boosted by CCL18 (a.k.a. DC-derived CC chemokine 1) [42]. It is interesting to note that blood stage forms of *P. yoelii* can act on DCs, which in turn can suppress the generation of cytotoxic T lymphocyte (CTL) responses against sporozoite re-infection in the liver [43]; however, these DCs can still trigger immune responses against further challenges with iRBCs [44]. Moreover, it has been shown that CD8α+ DCs (lymphoid DCs) are important in the induction of strong CD8+ T cell responses [45]. Thus, distinct DC types and parasite forms can trigger different immune responses against different forms of malaria parasites.

Some evidence suggests that Tregs participate in the control of DC potential. For example, immunization with recombinant circumsporozoite epitopes can trigger better protection against future infections if anti-CTLA-4 (which is present on Tregs) treatment is administered [46]. Also, an iRBC infection induces production of the pro-Treg cytokines TGF-β and IL-10 by DCs, which are close to T cells within the spleen [47].

Despite that these previous results suggest mechanisms for Tregs and DCs in the restriction of the immune system activation against experimental malaria infections, the precise roles of Tregs and DCs in the accumulation of parasites inside the liver were not measured; thus, we have addressed these issues in this paper. First, we have demonstrated the accumulation of *P. c*-iRBC within the liver through intravital microscopy and confirmed this by *in vitro* adhesion assays; next, we have characterized the modulatory roles of Tregs and DCs in the livers of *P. c. chabaudi*-infected mice; finally, we have analyzed the suppressive functions of Tregs that were isolated from the livers of *P. c. chabaudi*-infected mice. Our results support the hypothesis that the accumulation of *P. c*-iRBCs inside the liver can trigger suppressive events in this organ that favor parasite survival and the formation of a long-lasting parasite reservoir.

## Results

### *In vivo* parasite accumulation within the liver and liver pathology

Because the reporter mice used in this study were on a B10.PL background, we analyzed parasitemia curves in this mouse line and determined that parasite blood load was similar to B6 mice (Figure S1A). Thus, we assumed that B10.PL and B6 mice were similar in terms of resistance to *P. c. chabaudi* infection.

**Figure 1:**
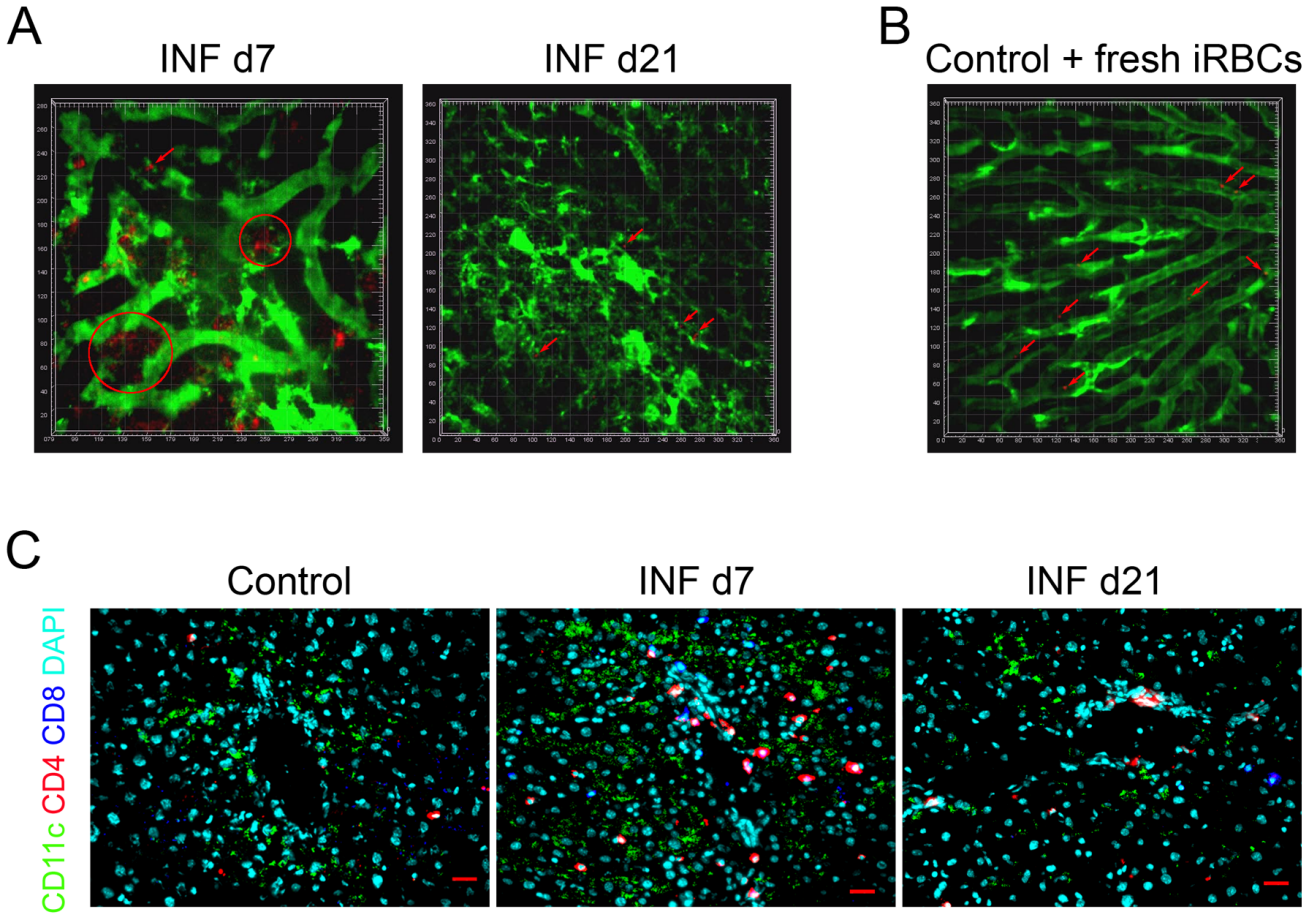
*In vivo* accumulation of iRBCs and leukocyte sequestration within the liver. B10.PL mice were intraperitoneally infected or not with Pc-AS-mCherry. All animals were divided into three groups: (A) infected, on day 7 of infection (INF d7); infected, on day 21 of infection (INF d21); and (B) Control (non-infected) mice that were freshly injected with iRBCs and immediately imaged. Each square area represents 20×20 µm. The red arrows indicate red-iRBC accumulation points within the liver blood vessels. Red circles represent foci of intense iRBC accumulation. To allow for the observation of blood vessels, FITC-Dextran was injected just after image acquisition. (C) Livers from Control, INF d7, and INF d21 mice were frozen, cut, and analyzed by IF. Green, red, blue, and DAPI colors correspond to CD11c, CD4, CD8, and DAPI staining, respectively. Red bar  = 20 µm. These results are representative of 3 repetitions.

Intravital images of livers on infection (INF) d7 revealed a massive accumulation of Pc-mCherry-labeled iRBCs that were in close contact with each other and with the sinusoidal walls, leading to the formation of iRBC aggregations and some rosettes along the sinusoidal vessels (Figure 1A and Video S1). To our knowledge, this is the first study that shows in vivo accumulation of iRBCs in the liver, as well as the forming of rosettes inside its blood vessels. Therefore, we hope these results will contribute to solve the issue of parasite accumulation inside the liver *in vivo*. It is also possible to observe transitory blood flow interruptions in some parts of the vessels (Video S1). On INF d21, the images still revealed iRBC adhesions, although these were reduced in comparison to INF d7 (Figure 1A and Video S2). This reduction is probably related to the differences in parasitemia levels between days 7 and 21 after infection (Figure S1A). Liver histological analysis from INF d7 mice showed an extensive mixed inflammatory infiltrate around the portal space, dispersed inflammation in sinusoidal vessels, adhesion of immune cells and iRBCs on LSEC, and damaged hepatocytes with nuclear fragmentation and cytoplasmatic vacuolization (Figure S2, A and B). Higher expression levels of endothelial receptor ICAM-1 were observed in the livers from the INF d7 group relative to Control (non-infected) and INF d21 groups (Figure S1B). No differences in CD36 expression were detected between the Control and infected groups (data not shown). The over-expression of ICAM-1 might be used by the iRBC and immune system cells for adherence to endothelial cells and LSECs. The *in vitro* cytoadhesion assay data corroborate this hypothesis because mature forms of iRBCs were found to adhere to INF d7 liver slices at higher levels than those for Control livers (Figure S1C, left) and to ICAM-1-transfected CHO cells (Figure S1C, right). Our immunofluorescence (IF) analysis confirmed the accumulation of immune cells around the portal space, with some cells spread through the liver parenchyma, in livers from INF d7 mice. We also observed accumulations of CD4+ T cells and DCs in these tissue locations (Figure 1C).

INF d21 livers returned to conditions similar to those in Control livers, as determined by decreases in tissue inflammatory infiltrates (Figure S2), hepatocyte damage (Figure S2), and cytoadhesion of iRBCs (Fig. 1A and Video S2) and immune system cells (Figure 1C) on sinusoidal walls.

Interestingly, the injection of synchronized mature forms of iRBCs into a normal non-infected B10.PL mouse revealed that iRBCs could adhere to sinusoidal walls (Figure 1B and Video S3), which expressed basal levels of endothelial receptors ICAM-1 (Figure S1B).

### Effects of parasite accumulation on DC and Treg behaviors

**Figure 2:**
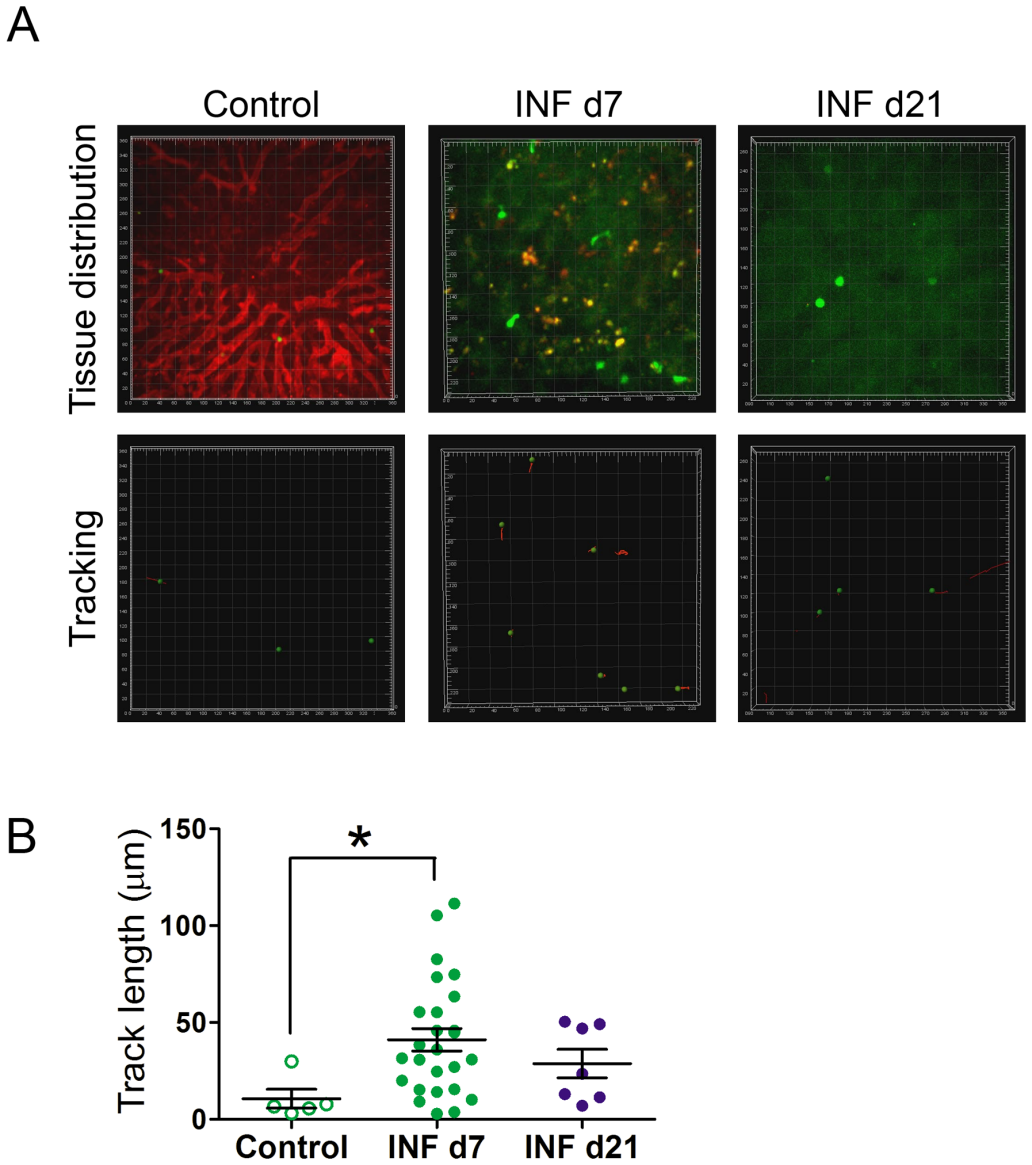
*In vivo* modulation of Treg behavior within the liver after infection with iRBCs. Foxp3-GFP mice were intraperitoneally infected or not with Pc-iRBCs. All animals were divided into three groups: (A) Control (non-infected); infected, on day 7 of infection (INF d7); infected, on day 21 of infection (INF d21). Each square area represents 20×20 µm. To allow for the observation of blood vessels, Rhodamine B-Dextran was injected just after image acquisition in Control mice. Green cells or spots represent Tregs, and red tracks indicate the movements of these cells. In the INF d7 group, note the high number of areas of iRBCs adhesion. (B) Distances crawled by Tregs (track length) from the groups mentioned above was measured. These results are representative of 3 repetitions.

Despite the observed liver injuries on INF d7, INF d21 livers returned to a near-normal state (Figure S2); the accumulation of DCs and CD4+ T cells within the INF d7 livers, as shown in ours IF (Figure 1C) raised the hypothesis that tolerogenic immunological mechanisms could be involved. Thus, we decided to evaluate whether two specific types of tolerogenic cells, Tregs and DCs, were present in these infiltrates and whether they could exert control against excessive inflammation. We initially evaluated Treg movement inside Control or infected livers (Figure 2A and B).

Intravital images of uninfected livers (Control group) showed Treg (Foxp3-GFP+ cells) circulation within the blood vessels with occasional pauses (Figure 2A and Video S4), in a manner similar to circulating leukocytes. In the INF d7 group, a different pattern occurred, which was characterized by an increased number of Tregs that were actively patrolling areas of cells with internalized iRBCs (Figure 2A and Video S5).

**Figure 3:**
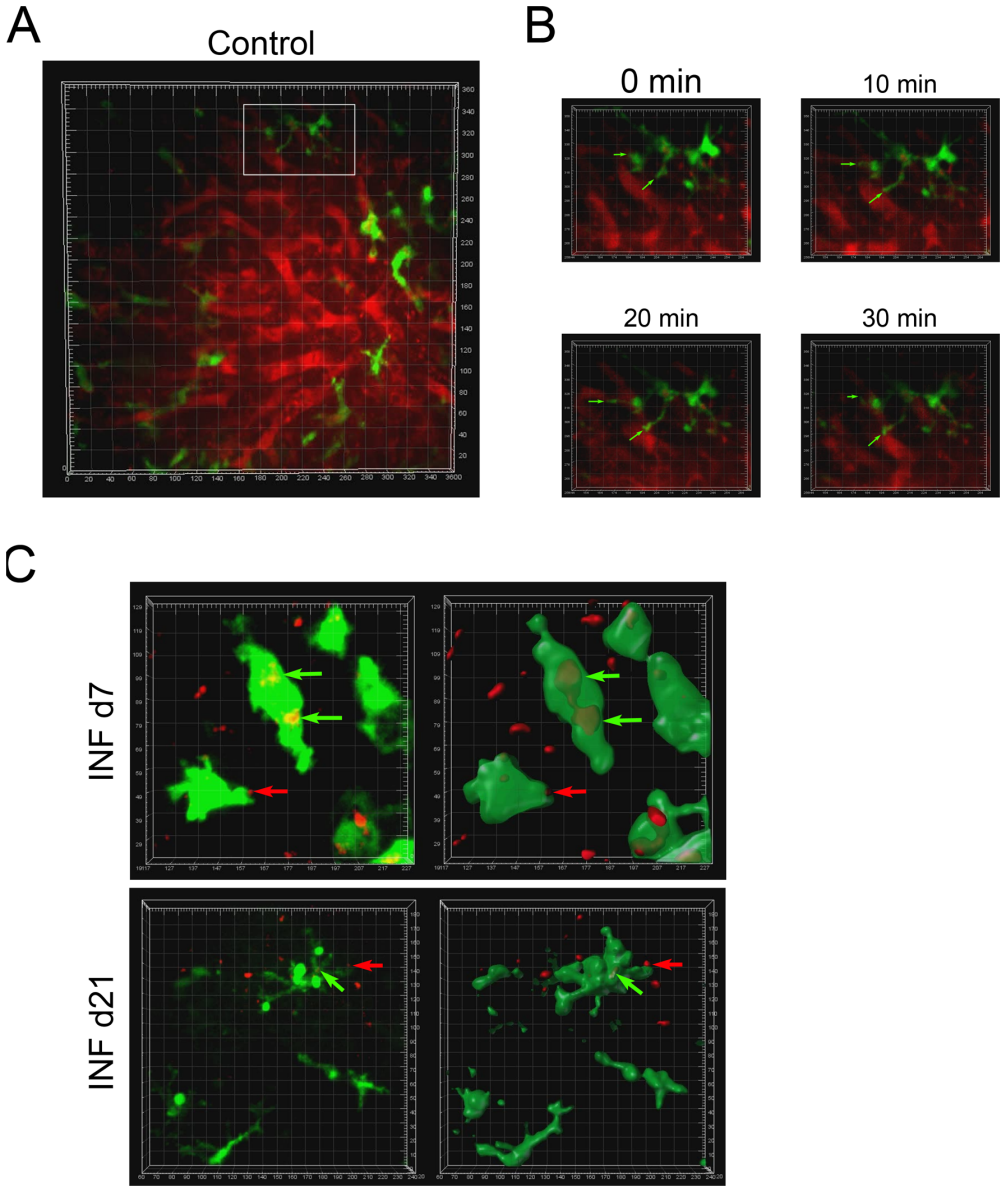
*In vivo* behavior of DCs within the liver before and after infection with iRBCs. CD11c-YFP mice were intraperitoneally infected or not with Pc-iRBCs. All animals were divided into three groups: (A and B) Control (non-infected); (C) infected, on day 7 of infection (INF d7); or infected, on day 21 of infection (INF d21). Each square area represents 20×20 µm. To allow for the observation of blood vessels, Rhodamine B-Dextran was injected just after image acquisition in Control mice. In (B), snapshots at different time-points reveal the dendrite behavior of some DCs within the Control liver. In (C), at left, the 3D distribution of DCs and iRBCs inside the infected livers; at right, a 3D model of these distributions in which DC models are represented in transparent-green to allow observation of red-iRBCs within them. Green arrows show DCs that had internalized iRBCs. Red arrows show iRBCs in close contact with DCs. These results are representative of 3 repetitions.

CD11c-YFP+ B10.PL uninfected mice (Control group) were submitted to intravital microscopy to allow visualization of normal liver DC behavior, which exhibit active probing movements with their dendrites within the blood vessels (Figure 3, A and B; Videos S6 and S7). When we observed animals in the INF d7 group, the DCs were found to be enlarged, having reduced probing movements and internalized iRBCs (Figure 3C and Video S8). The presence of immune system cell infiltrates inside the liver, as shown by HE and IF experiments, together with the intravital images of Foxp3-GFP+ and CD11c-YFP+ mice suggests that Tregs could be acting within the inflammatory infiltrates to modulate DC functions or other cells, such as KFCs, LSECs, or Teff cells. The behaviors of Tregs and DCs in INF d21 mice (Figure 2A and Figure 3C; Videos S9 and S10) were similar to those observed in Control livers (Videos S4, S6, and S7).

We also measured Treg crawling distances (track lengths) in Control, IFN d7, and INF d21 groups (Figure 2B). It was clearly observed that Treg track lengths were higher in INF d7 than Control groups, once again corroborating to the actively patrolling behavior of these INF d7-Tregs (Figure 2B). Despite no statistical difference was observed between Treg track lengths from INF d7 or INF d21 groups, it seems that Treg track lengths in INF d21 livers were closer to the values found to the Control group.

### Phenotype of leukocytes inside the liver during iRBC accumulation

Intravital images of Tregs from INF d7 group revealed that Tregs had a patrolling behavior within the livers of these animals in which Tregs seemed to seek regions with inflammatory loci (Figure 2A and Video S5). This behavior might be linked to a differential phenotypic profile of leukocytes inside these inflamed livers; thus, we decided to investigate this hypothesis by flow cytometry analysis.

Flow cytometry analysis showed increased numbers of all evaluated cell types in livers from INF d7 group (Figure S3), which supported the presence of an intense inflammatory immune infiltrate in INF d7 livers that was observed by intravital microscopy (Figure 2A), IF (Figure 1B), and histology (Figure S2).

**Figure 4:**
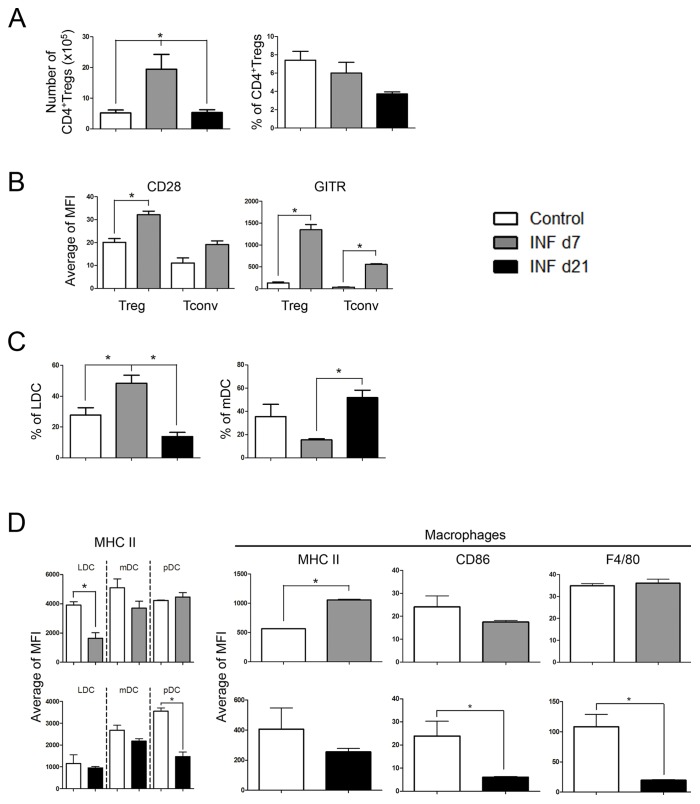
Infection with iRBCs induces alterations on Tregs and APCs inside the liver. Foxp3-GFP or CD11c-YFP mice were intraperitoneally infected or not with Pc-iRBCs. All animals were divided into three groups: Control (non-infected; open columns); infected, on day 7 of infection (INF d7; grey columns); or infected, on day 21 of infection (INF d21; black columns). Liver cells were stained with MAbs against a panel of surface molecules to identify different types and subtypes of leukocytes, as well as the expression levels of some proteins. (A) Numbers and percentages of Foxp3+CD4+ cell (Tregs) in the liver samples. (B) CD28 and GITR expression levels on Tregs. (C) Percentages of lymphoid (LDCs) or myeloid (mDCs) DCs in the liver samples. (D) MHCII, CD86, and F4/80 expression levels on DC subtypes and macrophages. These results are representative of 3 repetitions.

Treg numbers were increased in INF d7 group in comparison to Control or INF d21 groups (Figure 4A), and these cells expressed higher levels of CD28 and GITR than the other two groups (Figure 4B). No differences were observed in CTLA-4 or CD25 expression levels on INF d7 and INF d21 groups in comparison to Control group (Figure S4, B and C). Moreover, cells from INF d21 group had similar CD28 or GITR expression levels as cells from Control group (Figure S4C). Despite the finding that CD8+ Treg numbers in INF d7 group were higher than those in Control or INF d21 groups, these numbers were still quite lower than CD4 Treg numbers (Figure S4A *vs*. Figure 4A); double-positive (DP) CD4+CD8+ Treg numbers were similarly very low across all groups (Figure S4A).

Regarding antigen presenting cells (APCs), the frequency of lymphoid DCs (LDCs) increased and myeloid DCs (mDCs) decreased in INF d7 livers (Figure 4C) in comparison to Control and INF d21, whereas no differences were observed in the frequencies of plasmacytoid DCs (pDCs) (Figure S5A). The expression of MHCII molecules was decreased on LDCs from INF d7 group (Figure 4D) in comparison to those from the Control group; despite macrophages had an increase in the expression of MHC II molecules (Figure 4D), no differences in CD80 or CD86 expression were observed in either INF group (LDCs or macrophages) in comparison to Control group (Figures 4D and S5B). In the INF d21 group, decreased MHCII expression was observed on pDCs in comparison to the Control group (Figure 4D), while macrophages from INF d21 had similar MHCII expression levels but lower CD86 and F4/80 expression levels in comparison to the Control group (Figure 4D). No differences were observed in macrophage frequencies neither in CD80 expression in all tested times (Figure S5C).

In summary, flow cytometry analysis revealed alterations that were relative to the frequencies of APC subtypes (more LDCs than mDCs) and decreases in MHCII expression on LDCs from the INF d7 group. Decreases in MHCII expression levels on pDCs and in CD86 expression levels on macrophages were observed in the INF d21 group. These data, when associated with the active status of Tregs as measured by the over-expression of CD28 and GITR, suggest a functional suppressive role of Tregs against macrophages and dendritic cells. Tregs might control not only the frequencies of specific DC subsets but also the expression of molecules that are involved in optimal Ag-presentation, such as MHCII and B7 family costimulatory molecules, on DCs and macrophages.

### Proliferative capacities of conventional CD4+ T cells and suppressive capacities of Tregs from infected livers

**Figure 5:**
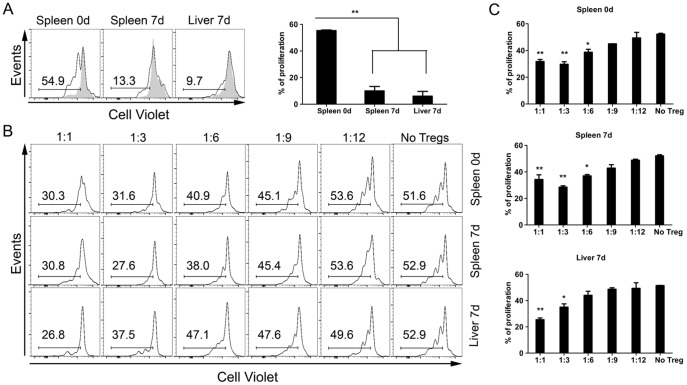
*In vitro* suppression capacity of liver Tregs after infection with iRBCs. Foxp3-GFP mice were intraperitoneally infected (or not) with Pc-iRBCs. All animals were divided into two groups: Control (non-infected) or infected, on day 7 of infection (INF d7). (A) Conventional CD4+Foxp3- T cells (Tconv) were isolated from INF d7 liver (Liver 7d) or spleen (Spleen 7d) samples or from non-infected spleens (Spleen 0d) by flow cytometry. Next, these cells were labeled with VioletCell tracer to measure proliferation by tracer dilution among cells. Note that Tconv from infected animals did not proliferate after *in vitro* anti-CD3 stimulation; proliferation was observed in non-infected Tconv only. (B) CD4+Foxp3+ T cells (Tregs) isolated from the same animals described above were co-cultured with APCs and naïve Tconv, with anti-CD3 stimulation. The histograms show the VioletCell tracer dilution after culture. (C) Percentages of naïve Tconv cells that proliferated with different concentrations of Tregs in culture. These results are representative of 3 independent experiments.

Thus far, it appeared that liver Tregs were active contributors to the suppression of immune responses in INF d7 mice. Next, we tested T cell proliferation and Treg suppressive capacities *in vitro* (Figure 5A and 5B, respectively).

CD4+ T cells that were isolated from livers of INF d7 mice exhibited a reduced proliferative capacity in comparison to CD4+ T cells that were isolated from spleens of Control animals as determined by CellTrace Violet dilution after 72 hours of *in vitro* culture (Figure 5A). Because nitric oxide (NO) has an important role in the suppression of CD4+ T cell proliferative responses in the acute phase of this model infection [48], we decided to measure the levels of iNOS mRNA in our liver samples by qtRT-PCR. As shown in Supplementary Fig. 6A, iNOS mRNA levels were higher in INF d7 group than Control group. This increase in iNOS expression, and consequently in NO secretion, primarily from macrophages, might trigger endothelial alterations that lead to vasodilatation and increased leukocyte infiltration as observed previously (Figure 1C, 2A, and Figure S2).

**Figure 6:**
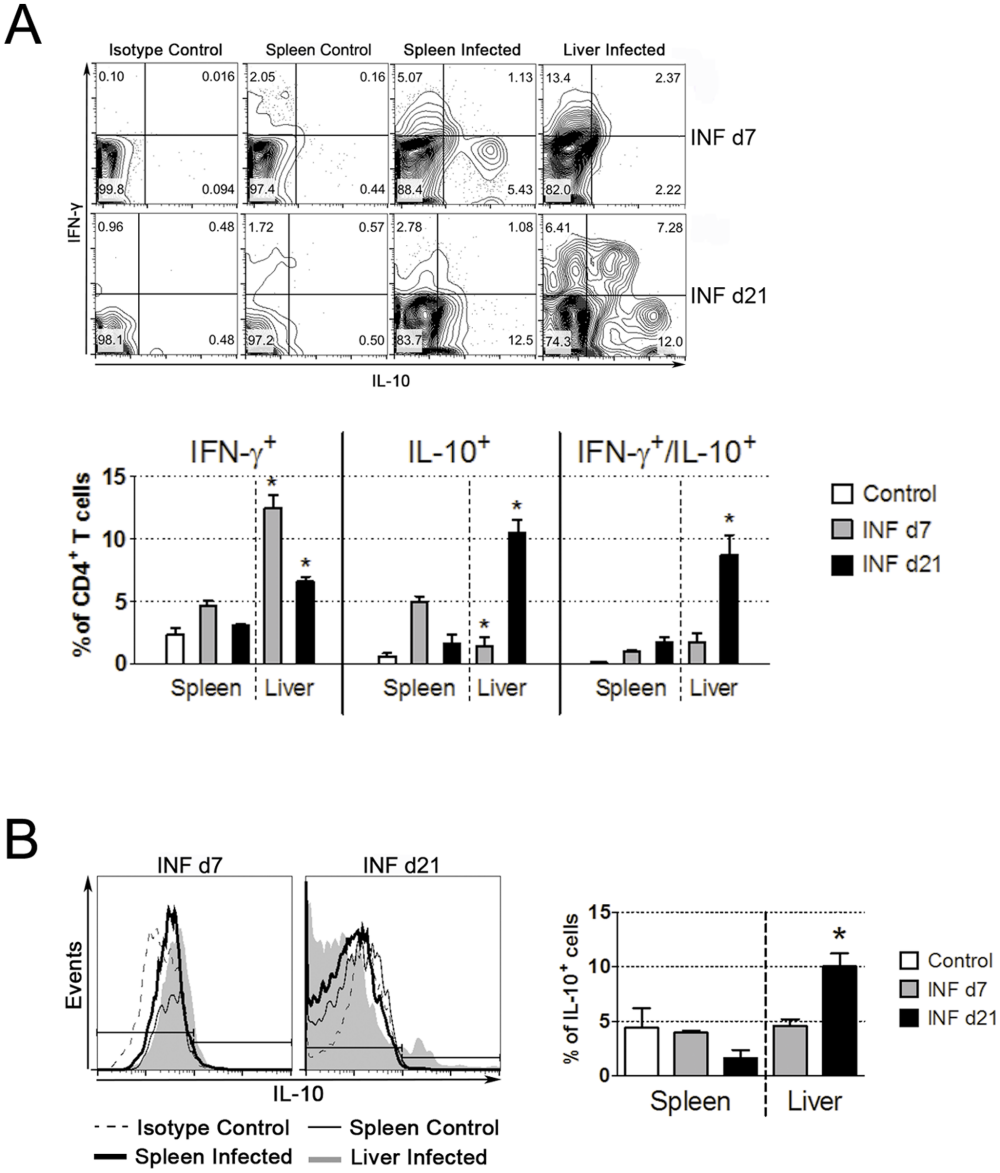
Infection with iRBCs leads to the increased production of IL-10 inside the liver. Foxp3-GFP mice were intraperitoneally infected or not with Pc-iRBCs. All animals were divided into three groups: Control (non-infected; open columns); infected, on day 7 of infection (INF d7; grey columns); or infected, on day 21 of infection (INF d21; black columns). Liver samples were processed and isolated cells were stained with MAbs against a panel of surface molecules to identify different types and subtypes of leukocytes, together with intracellular anti-cytokine staining. (A) IL-10 and IFN-γ production by CD4+ T cells. (B) IL-10 production by CD11c+ cells. These results are representative of three independent experiments.

Since IL-10 might also be responsible for the lack of proliferative capacity observed in CD4+ T cells from INF d7 livers, we measured the mRNA levels of this cytokine in liver samples and found a higher expression of IL-10 mRNA in INF d7 livers in comparison to Control livers (Figure S6B). Small percentages of IL-10-producing cells were found inside the liver CD4+ T cells on day 7 of infection (Figure 6A) and CD4+ T cells producing only IFN-γ were also found in these suspensions. We found similar percentages IL-10/IFN-γ double producer T cells in spleen or liver cell suspensions on INF d7 groups (Figure 6A). However, on day 21 after infection, the percentage of IFN-γ-producing CD4+ T cells decreased inside the liver and we found a considerable percentage of CD4+ T cells producing IL-10, including double IFN-γ/IL-10 producers (Figure 6A), and some IL-10-producing DCs (Figure 6B).

To test the liver-Treg suppressive capacity, we performed *in vitro* suppression assays and observed that INF d7 liver-Tregs are good suppressors of spleen CD4 naïve T cells (Figure 5B and 5C), with suppressive capacities similar to Tregs from Control or INF d7 spleens Tregs. Taken together, non-Treg CD4+ T cells from liver on day 7 of infection might be under the control of two different pathways: one that is iNOS/IL-10-dependent, to control resident and activated CD4+ T cells, and a second that is Treg-dependent, to control the newcomer CD4+ T cells.

## Discussion

The causes that lead to the preferential accumulation of parasites within the livers of *P. c. chabaudi* AS-infected mice are still obscure. Liver accumulation of iRBCs could be important for parasite survival by allowing the parasites to avoid immune system surveillance inside the spleen [4]. Once these iRBCs are trapped within the organ, different responses are triggered that lead to tissue damage and other pathological effects.

A considerable upregulation of *cir* gene expression occurs at the peak of parasitemia in *P. c. chabaudi* infection model [19]. At this peak, excessive intravascular hemolysis occurs and is responsible for an increased oxidative stress environment due to heme overload, which culminates in leukocyte infiltration and massive parasite accumulation within the liver as seen in *P. yoelli*–infected BALB/c mice. In this model, NF-kB activation is fundamental in the attraction of leukocytes to the liver and consequently to cause tissue damage [49]. NF-kB activation is favored by oxidative stress and leads to the expression of ICAM-1, VCAM-1, CXCL1, and CXCL2 in hepatocytes. Thus, neutrophils reach the liver parenchyma, trigger tissue damage, and generate more reactive oxidants, creating a damaging feedback loop (54). The expression of these molecules in the LSEC of *P. c. chabaudi* AS-infected mice could favor iRBC accumulation and infiltration of immune cells.

In our work, B10.PL mice reached a parasitemia peak on day 7 of infection (Figure S1A) and developed large liver areas with necrotic and apoptotic characteristics (Figure S2), and an intense infiltrate of CD4+ T cells and DCs close to the portal space (Figure 1C). Despite this intense initial inflammatory reaction, on day 21 of infection, a near-complete tissue recovery was observed, with few remaining necrotic areas (Figure S2). The observed weight loss in malaria-infected mice is in accordance with the generated acute inflammatory environment and reaches the lowest levels concomitant with the parasitemia peak (data not shown). Taken together, these initial results point to a very well-controlled inflammatory process in which tissue-protective counter measures are likely in place to curb excessive inflammation.

An interesting discussion can be raised about iRBC accumulation within the liver. It is unknown whether the parasite infection-driven liver inflammation triggers the increased expression of endothelial receptors, thus favoring increased iRBC adhesion, or if such basal expression of receptors is enough to interact with some iRBCs, which in turn would induce increased inflammation and increased expression of adhesion molecules and the formation of a positive adhesion feedback loop. We briefly tested this last hypothesis by injection of freshly isolated and synchronized mature forms of iRBCs into non-infected mice. Interestingly, we observed iRBC accumulation within the livers of these mice (Figure 1B and Video S3); thus, it appears that a subset of iRBCs expresses molecules that allow binding of these cells to basal level receptors expressed on endothelial cells.

Regarding liver leukocyte infiltration, the phenotypic analysis of immune cells agreed with the observed histological changes. The numbers of all analyzed cells increased on day 7 of infection and then returned to the non-infected levels on day 21 after infection (Figure S3). Despite this strong inflammatory response, which included high numbers of APCs (DCs and macrophages), CD4+ and CD8+ T cells, NK and NKT cells, we observed some interesting modulatory patterns of activation molecules on these cells. It is accepted that liver can promotes immune tolerance, with LSEC and KFCs actively participating in this process [22], [23]. Under normal conditions, these cells express low levels of MHCII and costimulatory molecules; moreover, they secrete IL-10 and PGE2 after LPS stimulation. Therefore, it is possible that different types of cells within the liver could trigger suppressive responses under pathological conditions.

In our analysis, the numbers of lymphoid, myeloid, and plasmacytoid DCs increased on day 7 of infection (Figure S3). However, it was interesting to note that lymphoid DC (LDC) frequencies were predominant during the acute phase of the infection (day 7) (Figure 4C), although these cells had decreased levels of MHCII molecules (Figure 4D). On day 21 of infection, only plasmacytoid DCs (pDCs) had decreased MHCII expression levels (Figure 4D), with no observed differences in the expression levels of costimulatory molecules (Figure. S5B).

Macrophages, despite their high numbers during the acute phase of infection, had similar levels of MHCII and costimulatory molecules when compared with control cells (Figure 4D). On day 21 of infection, decreases in the expression levels of CD86 and F4/80 were noted (Figure 4D). In general, decreases in the expression levels of MHCII and costimulatory molecules can contribute to a failure in the initiation and amplification of the adaptive immune response, which predispose for the generation of immune tolerance and parasite survival. Intravital images of infected CD11c-YFP mice revealed intense contacts between DCs and iRBCs (Video S8) and internalization of iRBCs (Figure 3C). Our IF experiments showed that DCs and CD4+ T cells were closely colocalized on day 7 of infection (Figure 1C), which might indicate a modulation of DC activation by some CD4+ T cell types, such as Tregs. In fact, we found high numbers of CD4+Foxp3+GFP+ Tregs within the liver on day 7 of infection (Figure 4A), which corroborates this hypothesis. These Tregs also expressed higher levels of CD28 and GITR, which indicated a more actived status of these Tregs as compared to similar cells from Control animals (Figure 4B).

The suppression assay experiment confirmed the inhibitory functional status of liver Tregs on conventional naïve CD4+ T cells from spleen (Figure 5B). Tregs that were isolated from liver during the acute phase of infection suppressed naïve CD4+ T cell proliferation, but did not suppressed the proliferation of liver effector CD4+ T cells (Teffs) from the acute phase. However, it is important to note that these Teffs did not show an intrinsic proliferation capacity *in vitro* at day 7 of infection, unlike that observed in CD4+ T cells from spleens of the same animals (Figure 5A). These observations suggest two possible mechanisms by which the inflammatory immune response is controlled within the liver: one mediated by CD4+ Tregs against the activation of newly arrived naïve CD4+ T cells at the inflammation *loci*, and other intrinsic to the inflammation that controls the proliferation of Teffs. Tregs might also modulate the Ag-presenting capacity of APCs through alterations in the expression of MHCII, CD80, and CD86 molecules [50].

Furthermore, a subtype of Tregs can be induced in the periphery by an iNOS-dependent manner, which in turn produces high levels of suppressor-type cytokines such as IL-10 [51]. The increased production of IL-10 mRNA in INF d7 livers, when compared to Control livers (Figure S6B), suggests that this Treg subtype might be present, although not visible in our intravital experiments because since they are Foxp3- [51]. It is also possible that LSEC and KFCs could be the source of IL-10, thus curbing the inflammatory response. Therefore, it is possible that iNOS and IL-10 could modulate alterations in the expression of MHCII and costimulatory molecules on APCs (Figure 4D). Our data shows that liver CD4 T cells and DCs were the source of IL-10 on day 21 of infection (Figure 6) and, surprisingly, on day 21 after infection (Figure 6), liver IL-10/IFN-γ double producer cells were one of the major IL-10-producing cells found, more than the percentages found inside the spleen, as found by us and other groups studying cytokine production inside the spleen after infection [52]. Therefore, it seems that highly activated IFN-γ+ Th1 cells are an important source of IL-10 to guarantee protection against severe immune-mediated pathology inside the liver, and they may have a more important role inside the liver than the spleen, or the spleen IFN-γ+ Th1 cells previously described [52] may fulfill their “*raison d'être*” migrating to the liver.

Another important aspect that points to a probable active functional status of Tregs was suggested by the images acquired on day 7 of infection. *In vivo*, Tregs form stable contacts with target cells while suppressing the immune response [53]. In our experiments, we observed that Tregs patrolled the sinusoids of the liver parenchyma, in liver areas where cells that had internalized iRBCs could be found (Figure 2 and Video S5). The images of infected livers on day 21 of infection showed fewer iRBCs and Tregs within the observed areas as well as the termination of the patrolling behavior (Figure 2 and Video S9). We believe these Tregs were seeking inflammatory *foci* to curb excessive inflammation and future tissue damage; however, this hypothesis needs to be further elucidated.

In conclusion, a better understanding of the iRBC accumulation phenomenon, through the discovery of parasite antigens that bind to endothelial receptors, is needed to further the development of vaccines or drugs that can block this condition that is often associated with death in human malaria patients. Our study analyzed other aspects that are linked to *P. c. chabaudi* AS-iRBC accumulation within the liver in an attempt to correlate the intense leukocyte accumulation during the parasitemia peak and the rapid tissue recovery to a normal state when the blood parasite levels are low.

**Figure 7:**
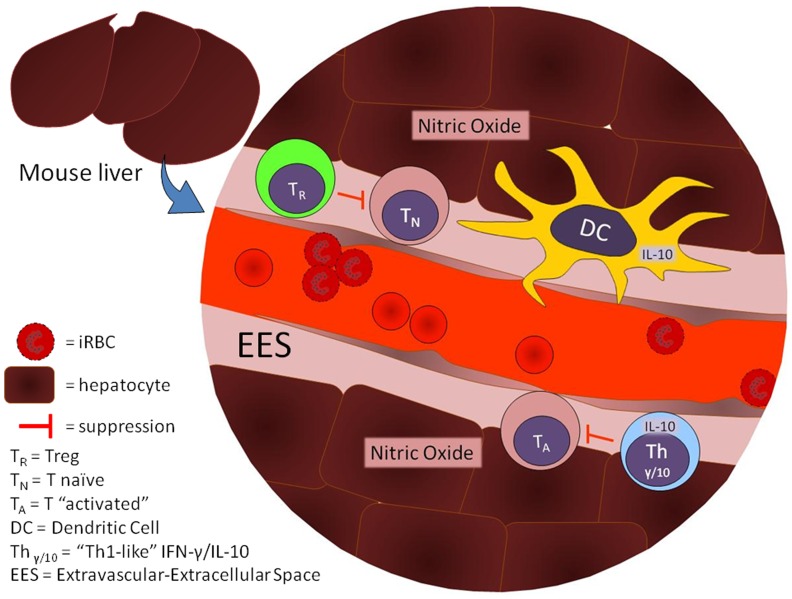
Hypothetical roles of Treg, Th1-like, DC, IL-10, and Nitric Oxide (NO) in the immuneregulation of liver damage. Foxp3-GFP Tregs (T_R_) would be responsible to control the activation of incoming naïve T cells (T_N_), while resident “activated” T cells (T_A_) cannot get activated by the local IL-10 and NO production. Possible IL-10 sources would be Th1-like T cells (Th_γ/10_ cells) and Dendritic Cells (DCs).

The results obtained suggest an active functional status of Tregs within the liver during the parasitemia peak and alterations in the expression levels of important antigen-presentation molecules by APCs, which could also be modulated by Tregs. The intravenous injection of mature forms of iRBCs into a control (non-infected) mouse and the presence of iRBCs along the sinusoidal walls indicate that there are molecules expressed in these iRBCs and at endothelial cells that allow this binding to occur; this initial iRBC adhesion could trigger an influx of inflammatory cells and, consequently, increase tissue inflammation. To curb an excessive inflammatory response, intrinsic NO and IL-10 production, together with high Treg function, could suppress leukocyte activation. Despite necessary to control excessive liver damage, the corollary consequences of this suppression would be the maintenance of iRBCs inside the liver, as a last parasite refuge from the immune system (summary in Figure 7). Liver participation in the development of immune tolerance to erythrocytic malarial forms and the Treg role in this important process had not been analyzed previously, though both are of potential importance in the development of sterile immunity against blood stages of malaria.

## Materials and Methods

### Mice and parasites

C57BL/10.PL (B10.PL) mice were one of the strains used in this study and they were the background line for Foxp3-KI^gfp/gfp^ (Foxp3-GFP) [54]. CD11c.YFP transgenic (CD11c-YFP) [55] and TCR alpha/beta KO (TCRabKO) mice were also used in B10.PL background. All animals were raised in the animal facility at Instituto Gulbenkian de Ciência (IGC), Portugal. TCRabKO mice were used as a source of APCs for our *in vitro* suppression assays, as described elsewere. C57BL/6 animals were used to perform *in vitro* adhesion assays after *P. c. chabaudi* AS infections. These animals were raised in the animal facility of the Department of Immunology, Institute of Biomedical Sciences, University of São Paulo, Brazil.

The generation of *Plasmodium chabaudi chabaudi* AS mCherry was reported previously [56]. In our experiments, we expanded the parasite *in vivo* by intraperitoneal infections in RAG2 KO mice. At the peak of parasitemia, blood samples from these animals were harvested and aliquots of 5×10^7^ iRBCs/ml were frozen. All animals were kept in an inverted-light cycle to allow the observation and use of mature forms in all of our experiments.

Groups of infected mice (Foxp3-GFP or CD11c-YFP) were intraperitoneally injected with 2.5×10^7^ iRBCs and used on day 7^th^ (INF d7) and 21^th^ (INF d21) after infection. Control animals were not infected.

### Ethics statement

All procedures were approved by the Institutional Animal Care and Use Committee (IACUC) of the IGC and were in agreement with the Federation of European Laboratory Animal Science Associations (FELASA) directives. The approval ID number of this project is AO10/2010.

### Phenotyping of liver-associated immune cells

Foxp3-GFP or CD11c-YFP female mice at 6 to 8 weeks of age were kept on an inverted light cycle for 3 weeks prior to infection. Mice were injected intraperitoneally (IP) or not with 2.5×10^7^ iRBCs from *P. c. chabaudi* AS-mCherry (Pc-AS-mCherry)-infected mice. All animals were divided into three groups: Control (non-infected); infected, on day 7 of infection (INF d7); or infected, on day 21 of infection (INF d21). Parasitemia was verified by Giemsa-stained peripheral blood smears, and weight loss was measured on alternate days.

To harvest liver-associated immune cells, each infected or control animal was submitted to blood perfusion with cold PBS via cardiac puncture. Livers were removed and dissociated by mechanical and enzymatic digestion. Briefly, each liver was cut into small pieces with sterile scissors and incubated in an HBSS medium solution with 0.5 mg/ml of collagenase H (Roche, USA), 2% FCS, 0.6% BSA, and 0.02 µg/ml DNase I at 37°C for 30 min. Cold digestion solution was added to the tubes to stop the enzymatic digestion. The solutions were passed through a 70 µm sterile cell strainer (Fisher Scientific Inc., USA) and centrifuged at 500×g for 10 min at 4°C. Liver cells were washed twice and centrifuged at 40×g for 2 min at 4°C for hepatocyte separation. The supernatants were removed to fresh tubes, centrifuged at 500×g for 10 min at 4°C, and resuspended in complete RPMI medium (RPMI medium containing 10% FCS, 2 mM Glutamine, Penicillin/Streptomycin [100 µg/ml each], and 0.05 M 2-mercaptoethanol).

The cell suspensions were labeled further with anti-CD3 PE, anti-CD4 PerCP, and anti-CD8 APC MAbs to measure the frequencies of CD4+, CD8+ or DP cells among the conventional (GFP-) or regulatory (GFP+) T cells. NK and NKT cells were identified by staining with anti-CD3 APC and anti-Pan NK PE antibodies. Activation markers for CD4+ conventional T cells (Tconv) or Tregs were identified by staining with biotinylated anti-CD28, anti-CTLA-4, anti-CD62L, and anti-GITR antibodies (and further incubation with different types of Streptavidin (SAv) described below), and the directly labeled MAbs anti-CD25 PE or anti-CD122 PE. Dendritic cell subsets were identified by the following MAb staining combinations: anti-CD11c PE+ and anti-CD11b A647+ cells were considered to be myeloid DCs (mDCs), anti-CD11c PE+ and anti-CD8 APC+ cells were considered to be lymphoid DCs (LDCs), and anti-CD11c PE+ and anti-B220 A647+ were considered to be plasmacytoid DCs (pDCs). Anti-RT1B PerCP cross-reacted with B10.PL MHC class II molecules (MHCII) and it was used to measure MHCII expression in the DC subsets. Biotinylated anti-CD80 and anti-CD86 were used to measure the expression of these costimulatory molecules on APCs. Macrophages were considered to be CD11c-CD11b+ cells, and CD11b- F4/80 FITC+ cells were considered to be KFCs. B cells were labeled with anti-CD19 PECy7. Unlabelled anti-CD16/CD32 (Fc block, 1 µl/sample) was added to each MAb labeling mix to avoid non-specific staining. SAv PE, PerCP, and FITC were used to reveal biotinylated antibody staining. All antibodies were obtained from eBioscience Inc. (San Diego, CA, USA). Approximately 5×10^5^ cells per mouse were stained with antibody mixes that were diluted 1∶100 in FACS staining buffer (PBS, 2% FCS, and 0.1% Azide) at 4°C for 30 min. After washing with FACS staining buffer, 1 µl of SAv antibody was added to each sample and the samples were incubated for 20 min at 4°C. Finally, all samples were resuspended in FACS staining buffer and analyzed by flow cytometry. We used a FACSCalibur cytometer (BD Biosciences Inc., USA) to measure the staining of our samples, and the results were analyzed with FlowJo software. All graphics were created with GraphPad Prism 5 software. Each experimental group was composed by 3 mice. The results shown were representative of 3 independent experiments.

CD4+Foxp3+ T cells were considered Tregs while CD4+Foxp3- T cells were considered conventional T cells (Tconv).

### Intracellular cytokine stainings

To evaluate the production of IFN-γ and IL-10 by spleen and liver cells, experimental mice were anesthetized, perfused, euthanized, and cells were processed as described before. After eliminating RBCs with ACK buffer, 5×10^6^ cells were incubated for 4 h in the presence of PMA (50 ng/ml) and Ionomycin (500 ng/ml), with posterior addition of Monensin (2 mg/ml) as Stop Golgi. After this period, cells were stained for extracellular markers (CD4, CD8, CD11c, F4/80, Gr1 – all antibodies were purchased from BD Biosciences Inc, USA) for 30 min at 4°C, and then fixed and permeabilized with Cytofix/Cytoperm kit accordingly to the manufacturer instructions (BD Biosciences Inc, USA). Finally, cells were stained with anti-IFN-γ and anti-IL-10 antibodies (from BD Biosciences Inc, USA), and analyzed in a FACSCalibur cytometer. The plots and gating analysis were made in FlowJo software (Treestar Inc., USA).

### Liver intravital images

Infected animals, as described previously, were imaged on days 7 (INF d7) or 21 (INF d21) after infection. Control animals (non-infected mice) were used as a reference for normal cell behavior. Images of livers from Control animals freshly infected with iRBCs started to be acquired to more than 3 min after parasite injection.

To perform surgeries, animals were anesthetized via IP injection of 1.2×10^-3^ g/mouse weight (g) of Ketamine (Imalgene 1000) and 1.6×10^−2^ mg/mouse weight (g) of Xylazine (Rompum 2%), and the hair on the upper left quadrant was removed with depilatory cream. For each surgery, the abdominal cavity was opened, the anterior surface of the liver left lobe was exposed, and the lobe was placed on top of a metal stand with a central hole that was covered by a coverglass slide (24×60 mm). FITC-Dextran (1 mg; Sigma-Aldrich Inc., USA) or Rhodamine B (1 mg; Sigma-Aldrich Inc., USA), diluted in 100 µl of PBS, was intravenously injected into each mouse. All surgeries were terminal and the animals were euthanized.

Images were acquired for 20 to 45 minutes with an Andor Revolution XD spinning disk confocal microscope (Intelligent Imaging Innovations, Inc., USA) at 37°C. A 20X objective with a working distance of 1 mm was used in all acquisitions. A 1.5X additional magnification was used in some of our movies. The depth of each z-volume was divided into 7 to 10 z-stacks, with 2 µm between consecutive stacks. Images were analyzed by FIJI software (General public license, NIH, USA) and IMARIS software (Bitplane AG Inc., USA).

### Immunofluorescence staining

Liver samples were harvested from mice that had been submitted to intravital imaging, as described above, frozen in Tissue-Tek®O.C.T. ™ (Sakura Finetek, The Netherlands), and stored at −20°C. Liver slices were cut at a thickness of 0.8 µm with a Leica CM3050S Cryostat machine (Leica Inc., USA). To prepare the slices for IF staining, each slide with slices was fixed with 1% paraformaldehyde in PBS, pH 7.6, for 30 min at room temperature (RT). Excess O.C.T. was removed, and the slices were washed 3 times with PBS for 3 minutes at RT. Blocking solution (Fcblock and BSA 10%) was added for 1 h at RT, followed by 3 more washes with PBS. anti-CD3 APC, anti-CD4 PerCP, and anti-CD8 APC MAbs and biotinylated anti-CD11c Ab, diluted 1∶300, were applied to each liver slice. The samples were incubated for 1 h at 4°C in a humidified chamber. After 3 more washes with PBS, SAv-FITC or SAv-APC, diluted 1∶300, was added and the samples were incubated under the previous conditions. After 3 more washes with PBS, DAPI (5 µg/ml) was added for 5 min at RT, followed by 3 more washes. Fluoromount G (SouthernBiotech Inc., USA) was added to each slice, and each slide was covered with a coverglass. Finally, the samples were observed in a Leica DM RA2 microscope (Leica Inc., USA). We did not find any background staining with isotype control MAbs or SAv incubation without primary antibodies.

### Histopathology analysis

Liver samples were cut immediately after perfusion and were fixed in 4% buffered formalin (Sigma-Aldrich Inc., USA) for 24 h. Each sample was embedded in paraffin and cut into 5 µm slices. Next, slides with 3 slices each were submitted to haematoxylin-eosin staining and mounted with Permount mounting medium (Fischer Scientific Inc., USA). All images were acquired in a Leica DM LB2 microscope (Leica Inc., USA). Each tissue section was evaluated for the presence of cellular infiltrates, edema, hepatocyte vacuolization, hemozoin deposits, and iRBCs.

### Transcription analysis of IL-10, iNOS, ICAM-1, and CD36

Total RNA from the individual livers of non-infected (Control), 7 day- (INF d7), or 21 day-infected (INF d21) mice was obtained using an RNeasy Mini Kit (Qiagen Inc., USA), following the manufacturer's instructions for animal tissues. One microgram of total RNA was converted to cDNA (Transcriptor First Strand cDNA Synthesis Kit, Roche) using random hexamer primers. IL-10, iNOS, CD36, and ICAM-1 expression was quantified with the Applied Biosystems Power SYBR Green PCR Master Mix. IL-10 (il-10), iNOS (inos), CD36 (Cd36), ICAM-1 (Icam1), and HPRT (Hprt) specific primer sequences were as follows: il-10, 5′ –AAG GAC CAG CTG GAC AAC AT-3′ and 5′ –TCA TTT CC GAT AAG GCT TGG-3′; inos 5′ –CAG CTG GGC TGT ACA AAC CTT-3′ and 5′ –CAT TGG AAG TGA AGC GTT TCG-3′; Cd36, 5′ –TGG AGC TGT TAT TGG TGC AG– 3′ and 5′ –TGG GTT TTG CAC ATC AAA GA-3′; Icam1, 5′ – CGA AGG TGG TTC TTC TGA GC- 3′ and 5′ –GTC TGC TGA GAC CCC TCT TG-3′; Hprt, 5′-TGC TCG AGA TGT GAT GAA GG-3′ and 5′ – TCC CCT GTT GAC TGG TCA TT-3′; and Beta-actin, 5′- CCTGAACCCTAAGGCCAAC-3′ and 5-GCCTGGATGGCTACGTACA-3′. The gene expression quantification reactions were performed according to the manufacturer's instructions on an Applied Biosystems 7500 Fast Real-Time PCR System. All results were normalized according to the constitutive expression of the HPRT gene and were quantified with the 2ΔΔCT method.

### Cell proliferation assay

Foxp3 GFP female mice, 6 to 8 weeks of age, were infected as described previous and livers and spleens cells isolated as described in “***Phenotyping of liver-associated immune cells*** *”*. Tconv cells were isolated from liver and spleen labeled cells by flow cytometry (FACSAria, BD Biosciences, USA), using MAbs and labeling protocols described in “**Phenotyping of liver-associated immune cells**”. After sorting, purified cells were incubated for 10 min at 37°C, in PBS without fetal calf serum (FCS), with CellTrace Violet (Invitrogen Inc., USA) at 1 µM. After this incubation, extracellular dye was neutralized by the addition of FCS at final concentration of 10%, and cells centrifuged by 5 min, 400×g, at 4°C. 10^6^ purified liver or spleen Tconv were ressuspended in complete RPMI medium, and incubated with anti-CD3 (1 µg/ml) and anti-CD28 (0.5 µg/ml) MAbs, for 72 h, at 37°C, in a humidified cell culture incubator. The proliferation levels were measured by the CellTrace Violet dilution, in comparison with non-stimulated cells, where dye dilution is minimal or does not occur.

### Suppression assay

Foxp3-GFP-purified Tregs and Tconv (responder cells) from livers and spleens of infected day 7 or non-infected mice were FACSAria-sorted. Proliferation assays were set up in 96-well, round-bottom plates and contained, per well, 1×10^4^ responder cells, 2×10^4^ APCs (γ-irradiated spleen cells from TCRabKO mice), and anti-CD3 Ab at a concentration of 0.5 µg/ml. Putative Tregs were cocultured at Treg:responder ratios of 1∶1, 1∶3, 1∶6, 1∶9, 1∶12, and 0∶1 (No Treg). Proliferation was determined by CellTrace Violet dilution in the responder cells, on the third day of culture, in a flow cytometer. Two different types of Tregs were purified from infected mice: Liver 7d and Spleen7d. Their suppressive capacities were compared with Tregs from spleens of non-infected mice (Spleen 0d). Five Foxp3-GFP infected mice, 2 uninfected mice, and one TCR αβ KO were used in each experiment. The procedures were performed in 3 independent experiments.

### Enrichment of parasitized erythrocytes

To obtain mature forms (trophozoites/schizonts), red blood cells were collected from infected SCID mice with *P. chabaudi* AS and with 30% parasitemia by cardiac puncture and maintained in culture medium RPMI 1640 (Gibco Inc., USA). The erythrocytes were then enriched by magnetic separation column (MAC BEADS, Miltenyi Biotec, USA), resulting in cell populations with approximately 95% of iRBC.

### Cytoadherence assays

To verify the capacity of erythrocytes infected with *P. chabaudi* AS to adhere to hepatic tissue, synchronized and enriched iRBC (trophozoites/schizonts) were incubated on paraffin-embedded liver sections. Fifty microliters of enriched iRBC suspension, at the concentration of 108/ml, were overlaid on paraffin-embedded liver sections for 60 minutes at 37°C in a humid chamber. After washing the unbound cells, the slides were mounted with Vectashield contained DAPI (Vector Lab., Bruglingame, CA, USA) and examined under fluorescence microscopy (magnification 630X). The number of iRBC adhering to liver sections was determined in a blind fashion, counting 10 fields in each section. The second step was to evaluate the capacity of these iRBC to adhere to specific receptors. Initially iRBC were incubated with stable transfectants of CHO expressing CD36 [57] (CHO-CD36 cells) or Intercellular Adhesion Molecule 1 (ICAM-1) [58] (CHO-ICAM-1 cells) or with Control cells (CHO variant pgsA – CHO-745) [59] that not express CD36 or ICAM-1 for 1 h at 37°C in atmosphere containing 5% CO_2_. After washing, the cultures were fixed and stained with staining kit for blood count differential (Instant- Prov, New Prov Brazil). For the quantification of cytoadherence it was calculate the ratio between the number of CHO cells and the iRBC attached to them (n. iRBC attached/n. CHO cell).

### Statistical Analysis

Two-way ANOVA and Student's t tests were performed to determine significant differences between the groups in our experimental study. Our results represent the average and standard deviation of our samples. These tests yielded p values <0.05, which confirmed the statistical significance of our results.

## Supporting Information

Figure S1 **Parasitemia and in vitro adhesion of iRBCs.** B10.PL/B6 mice were intraperitoneally infected or not with Pc-iRBCs. (A) Parasitemia curve of infected-B10.PL/B6 mice. (B) Some animals were divided into three groups: Control (non-infected); infected, on day 7 of infection (INF d7); or infected, on day 21 of infection (INF d21) and mRNA expression levels measured by RealTime-PCR. (C) Adhesion analysis of iRBC in liver slices (left) or ICAM-1-transfected CHO cells (right). “Control” corresponds to non-transfected CHO cells. These results are representative of 3 repetitions.
(TIF)

Figure S2 **Liver physiological changes caused by infection with iRBCs.** B10.PL mice were intraperitoneally infected or not with Pc-iRBCs. All animals were divided into three groups: Control (non-infected); infected, on day 7 of infection (INF d7); or infected, on day 21 of infection (INF d21). (A) H&E staining of liver slices, showing hepatocytes (green arrows) in different conditions (inserts show these selected hepatocytes in detail), infiltration around the portal veins, and hemorrhage (*, INF d7). (B) H&E staining of liver slices, showing leukocyte adhesion to liver blood vessels (*, INF d7), perivascular infiltration (surrounded in black), and haemozoin deposition (green arrows). Black scale bar  = 40 µm. These results are representative of 3 repetitions. The inserts *i, ii, iii* show hepatocytes from Control, INF d7, and INF d21 groups, respectively.
(TIF)

Figure S3 **Numbers and phenotypes of liver leukocyte infiltrates after infection with iRBCs.** Foxp3-GFP or CD11c-YFP mice were intraperitoneally infected or not with Pc-iRBCs. All animals were divided into three groups: Control (non-infected; open columns); infected, on day 7 of infection (INF d7; grey columns); or infected, on day 21 of infection (INF d21; black columns). Liver cells were stained with MAbs against a panel of surface molecules to identify different types and subtypes of leukocytes. Total liver cell numbers were counted in a haemocytometer chamber, and the final numbers at each infection time were corrected according to the measured percentages. These results are representative of 3 repetitions.
(TIF)

Figure S4 **Infection with iRBCs and expression of cell surface molecules on liver Tregs and Tconv.** Foxp3-GFP mice were intraperitoneally infected (or not) with Pc-iRBCs. All animals were divided into three groups: Control (non-infected; open columns); infected, on day 7 of infection (INF d7; grey columns); or infected, on day 21 of infection (INF d21; black columns). Liver cells were stained with MAbs against a panel of surface molecules to identify different types and subtypes of leukocytes, as well as the expression levels of some proteins. (A) Percentages and numbers of Foxp3+CD4+CD8+ cells (DP Tregs) or Foxp3+CD8+ cells (CD8+ Tregs) inside the liver samples. (B) CD25 and CTLA-4 expression levels in CD4+ Tregs. (C) CD28, GITR, CD25, and CTLA-4 expression levels in CD4+ Tregs. These results are representative of 3 repetitions.
(TIF)

Figure S5 **Infection with iRBCs and expression of cell surface molecules on APCs inside the liver.** CD11c-YFP mice were intraperitoneally infected or not with Pc-iRBCs All animals were divided into three groups: Control (non-infected; open columns); infected, on day 7 of infection (INF d7; grey columns); or infected, on day 21 of infection (INF d21; black columns). Liver cells were stained with MAbs against a panel of surface molecules to identify different types and subtypes of leukocytes as well as the expression levels of some proteins. (A) Percentages of plasmacytoid DCs (pDCs). (B) CD80 and CD86 expression levels in different subtypes of DCs. (C) Percentage of macrophages and CD80 expression levels. These results are representative of 3 repetitions.
(TIF)

Figure S6 **Infection with iRBCs leads to the increased production of mRNA to iNOS and IL-10 inside the liver.** Foxp3-GFP mice were intraperitoneally infected or not with Pc-iRBCs. All animals were divided into three groups: Control (non-infected; open columns); infected, on day 7 of infection (INF d7; grey columns); or infected, on day 21 of infection (INF d21; black columns). Liver samples were frozen to further RT-PCR experiments. (A) iNOS mRNA levels. (B) IL-10 mRNA levels. These results are representative of three independent experiments.
(TIF)

Video S1 ***In vivo*** **adhesion of** ***P. chabaudi*** **-iRBCs in liver blood vessels during the parasitemia peak.** B10.PL mice were infected with Pc-iRBCs. On day 7 after infection (INF d7), each animal was submitted to a small surgery to expose one of the liver lobes. This lobe was imaged in a Spinning Disk confocal microscope for around 45 min. To allow the observation of blood vessels, each animal received FITC-Dextran i.v. just before image acquisition. Each square area corresponds to a 20×20 µm region. This movie is representative of 3 observed animals.
(AVI)

Video S2 ***In vivo*** **adhesion of** ***P. chabaudi*** **-iRBCs in liver blood vessels after the parasitemia peak.** B10.PL mice were infected with with Pc-iRBCs. On day 21 after infection (INF d21), each animal was submitted to a small surgery to expose one of the liver lobes. This lobe was imaged in a Spinning Disk confocal microscope for around 30 min. To allow the observation of blood vessels, each animal received FITC-Dextran i.v. just before image acquisition. Each square area corresponds to a 20×20 µm region. This movie is representative of 3 observed animals.
(AVI)

Video S3 ***In vivo*** **adhesion of** ***P. chabaudi*** **-iRBCs in liver blood vessels of non-infected mice.** B10.PL mice were injected with freshly isolated Pc-iRBCs and immediately after one of the liver lobes was imaged in a Spinning Disk confocal microscope for around 30 min. To allow the observation of blood vessels, each animal received FITC-Dextran i.v. just before image acquisition. Each square area corresponds to a 20×20 µm region. This movie is representative of 3 observed animals.
(AVI)

Video S4 **Intravital imaging of Foxp3-GFP non-infected (Control) liver.** Foxp3-GFP mice were submitted to a small surgery to expose one of the liver lobes. These lobes were imaged in a Spinning Disk confocal microscope for around 30 min. To allow the observation of blood vessels, each animal received Rhodamine B-Dextran i.v. just before image acquisition. Each square area corresponds to a 20×20 µm region. On the left side, a video showing the distribution and movement of Tregs; on the right side, a representation of Tregs (green dots) and their tracks (red lines). This movie is representative of 3 observed animals.
(AVI)

Video S5 **Intravital imaging of Foxp3-GFP INF d7 liver.** Foxp3-GFP mice were infected with Pc-iRBCs. On day 7 after infection (INF d7), each animal was submitted to a small surgery to expose one of the liver lobes. This lobe was imaged in a Spinning Disk confocal microscope for around 30 min. Each square area corresponds to a 20×20 µm region. On the left side, a video showing the distribution and movement of Tregs; on the right side, a representation of Tregs (green dots) and their tracks (red lines). Note that liver blood vessels, despite not labeled, can be seen by the background fluorescent noise of hepatocytes surrounding them. This movie is representative of 3 observed animals.
(AVI)

Video S6 **Intravital imaging of CD11c-YFP non-infected (Control) liver.** CD11c-YFP mice were submitted to a small surgery to expose one of the liver lobes. These lobes were imaged in a Spinning Disk confocal microscope for around 30 min. To allow the observation of blood vessels, each animal received Rhodamine B-Dextran i.v. just before image acquisition. Each square area corresponds to a 20×20 µm region. This movie is representative of 3 observed animals.
(AVI)

Video S7 **Intravital imaging of CD11c-YFP non-infected (Control) liver, detail.** The same movie as described at Supplementary Video 6, but now showing in more detail, one of the liver areas containing DCs actively probing the blood vessels with small dendrites.
(AVI)

Video S8 **Intravital imaging of CD11c-YFP INF d7 liver.** CD11c-YFP mice were infected with *Pc*-iRBCs. On day 7 after infection (INF d7), each animal was submitted to a small surgery to expose one of the liver lobes. These lobes were imaged in a Spinning Disk confocal microscope. Each square area corresponds to a 20×20 µm region. On the left side, a video showing the 3D distribution of DCs, where iRBCs can be found inside these cells; on the right side, a 3D model representation of these DCs (transparent green) and the iRBCs inside of them (red dots). Note that there are also iRBCs outside the DCs that are probably attached to liver blood vessels. This movie is representative of 3 observed animals.
(AVI)

Video S9 **Intravital imaging of Foxp3-GFP INF d21 liver.** Foxp3-GFP mice were infected with Pc-iRBCs. On day 21 after infection (INF d21), each animal was submitted to a small surgery to expose one of the liver lobes. This lobe was imaged in a Spinning Disk confocal microscope for around 30 min. Each square area corresponds to a 20×20 µm region. On the left side, the video showing the distribution and movement of Tregs; on the right side, a representation of Tregs (green dots) and their tracks (red lines). Note that liver blood vessels, despite not labeled, can be seen by the background fluorescent noise of hepatocytes surrounding them. This movie is representative of 3 observed animals.
(AVI)

Video S10 **Intravital imaging of CD11c-YFP INF d21 liver.** CD11c-YFP mice were infected with Pc-iRBCs. On day 21 after infection (INF d21), each animal was submitted to a small surgery to expose one of the liver lobes. These lobes were imaged in a Spinning Disk confocal microscope. Each square area corresponds to a 20×20 µm region. On the left side, a video showing the 3D distribution of DCs, where iRBCs can still be found inside of few DCs, but the majority were non-infected; on the right side, a 3D model representation of these DCs (transparent green) and the iRBCs inside of them (red dots). Note that there are also iRBCs outside the DCs that are probably attached to liver blood vessels. This movie is representative of 3 observed animals.
(AVI)
