# Supplementary figures and images for: Liver Accumulation of Plasmodium chabaudi-Infected Red Blood Cells and Modulation of Regulatory T Cell and Dendritic Cell Responses
Source: PLoS One. 2013 Nov 27;8(11):e81409. doi: 10.1371/journal.pone.0081409 (PMC3842419; doi:10.1371/journal.pone.0081409)

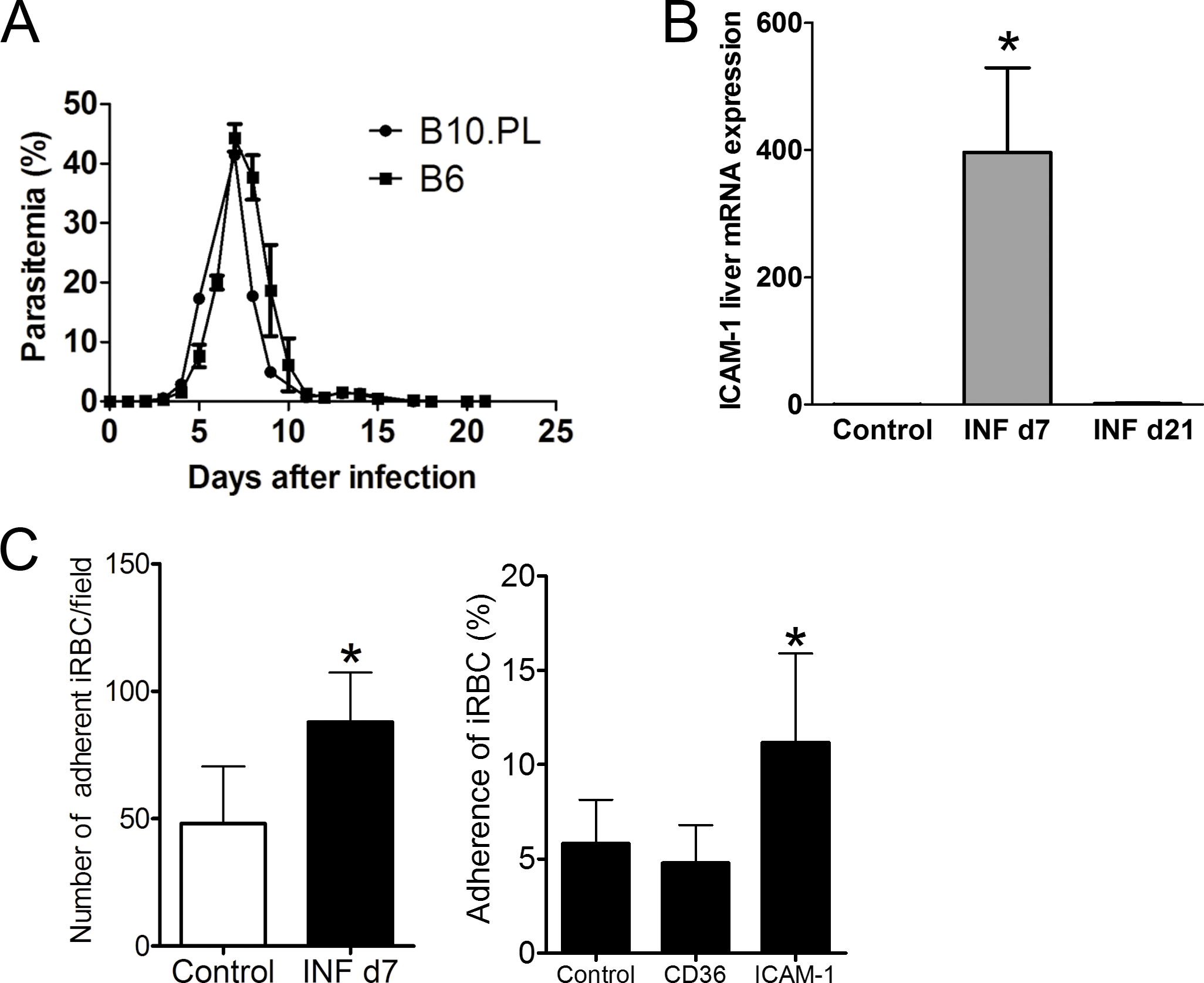

Supplement: Figure S1 — Parasitemia and in vitro adhesion of iRBCs. B10.PL/B6 mice were intraperitoneally infected or not with Pc-iRBCs. (A) Parasitemia curve of infected-B10.PL/B6 mice. (B) Some animals were divided into three groups: Control (non-infected); infected, on day 7 of infection (INF d7); or infected, on day 21 of infection (INF d21) and mRNA expression levels measured by RealTime-PCR. (C) Adhesion analysis of iRBC in liver slices (left) or ICAM-1-transfected CHO cells (right). “Control” corresponds to non-transfected CHO cells. These results are representative of 3 repetitions. (TIF) [file pone.0081409.s001.tif]

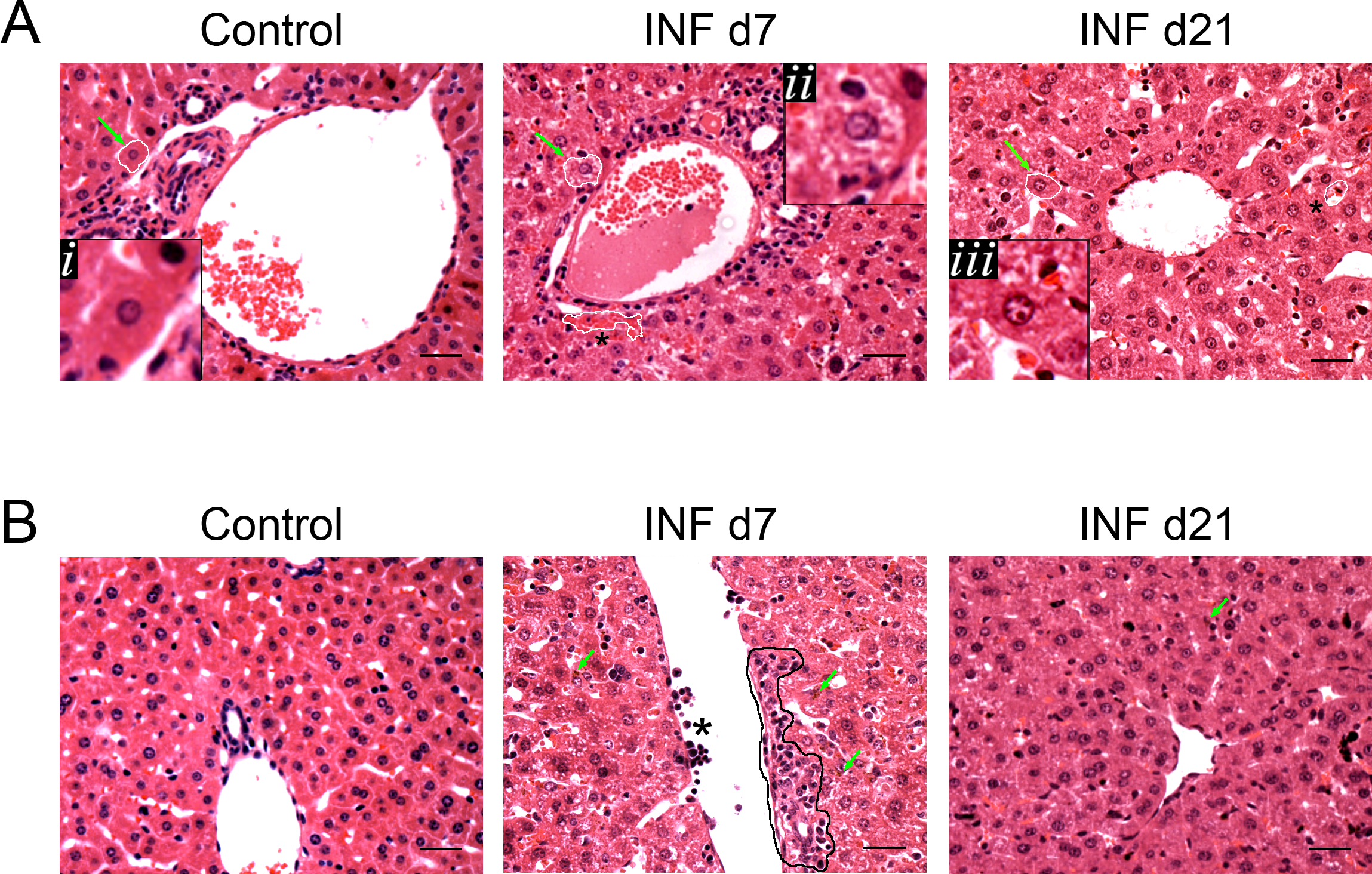

Supplement: Figure S2 — Liver physiological changes caused by infection with iRBCs. B10.PL mice were intraperitoneally infected or not with Pc-iRBCs. All animals were divided into three groups: Control (non-infected); infected, on day 7 of infection (INF d7); or infected, on day 21 of infection (INF d21). (A) H&E staining of liver slices, showing hepatocytes (green arrows) in different conditions (inserts show these selected hepatocytes in detail), infiltration around the portal veins, and hemorrhage (*, INF d7). (B) H&E staining of liver slices, showing leukocyte adhesion to liver blood vessels (*, INF d7), perivascular infiltration (surrounded in black), and haemozoin deposition (green arrows). Black scale bar = 40 µm. These results are representative of 3 repetitions. The inserts i, ii, iii show hepatocytes from Control, INF d7, and INF d21 groups, respectively. (TIF) [file pone.0081409.s002.tif]

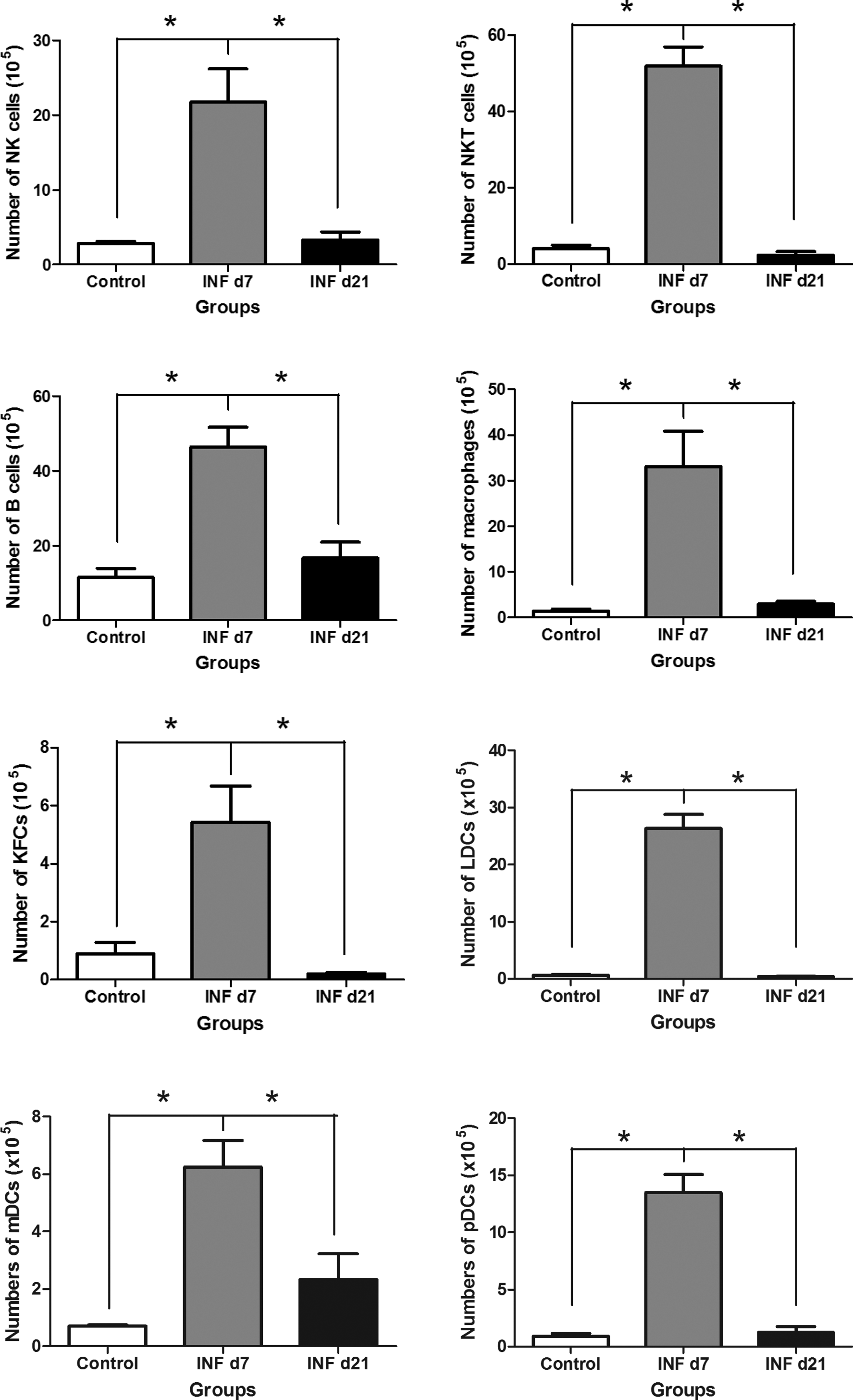

Supplement: Figure S3 — Numbers and phenotypes of liver leukocyte infiltrates after infection with iRBCs. Foxp3-GFP or CD11c-YFP mice were intraperitoneally infected or not with Pc-iRBCs. All animals were divided into three groups: Control (non-infected; open columns); infected, on day 7 of infection (INF d7; grey columns); or infected, on day 21 of infection (INF d21; black columns). Liver cells were stained with MAbs against a panel of surface molecules to identify different types and subtypes of leukocytes. Total liver cell numbers were counted in a haemocytometer chamber, and the final numbers at each infection time were corrected according to the measured percentages. These results are representative of 3 repetitions. (TIF) [file pone.0081409.s003.tif]

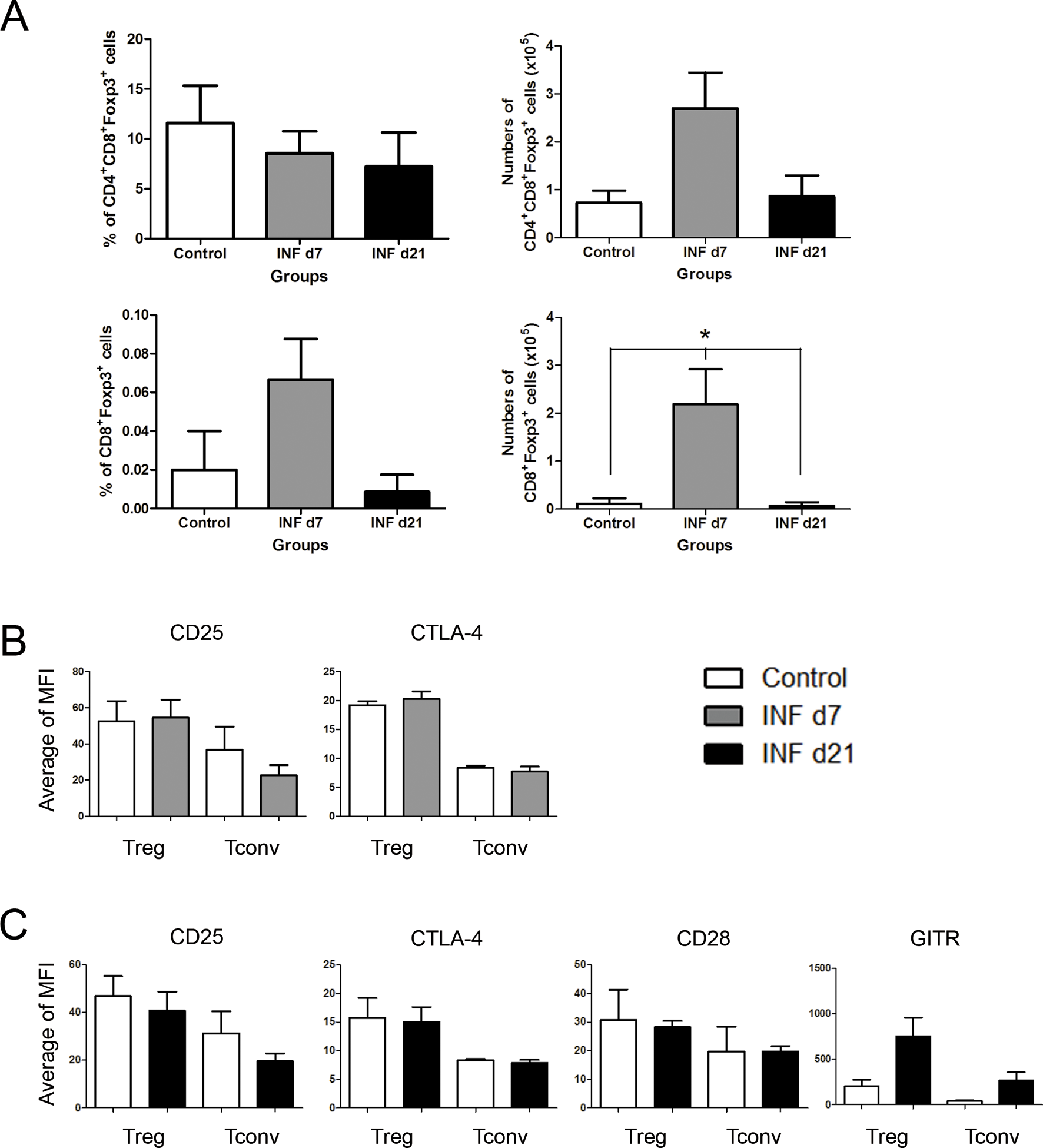

Supplement: Figure S4 — Infection with iRBCs and expression of cell surface molecules on liver Tregs and Tconv. Foxp3-GFP mice were intraperitoneally infected (or not) with Pc-iRBCs. All animals were divided into three groups: Control (non-infected; open columns); infected, on day 7 of infection (INF d7; grey columns); or infected, on day 21 of infection (INF d21; black columns). Liver cells were stained with MAbs against a panel of surface molecules to identify different types and subtypes of leukocytes, as well as the expression levels of some proteins. (A) Percentages and numbers of Foxp3+CD4+CD8+ cells (DP Tregs) or Foxp3+CD8+ cells (CD8+ Tregs) inside the liver samples. (B) CD25 and CTLA-4 expression levels in CD4+ Tregs. (C) CD28, GITR, CD25, and CTLA-4 expression levels in CD4+ Tregs. These results are representative of 3 repetitions. (TIF) [file pone.0081409.s004.tif]

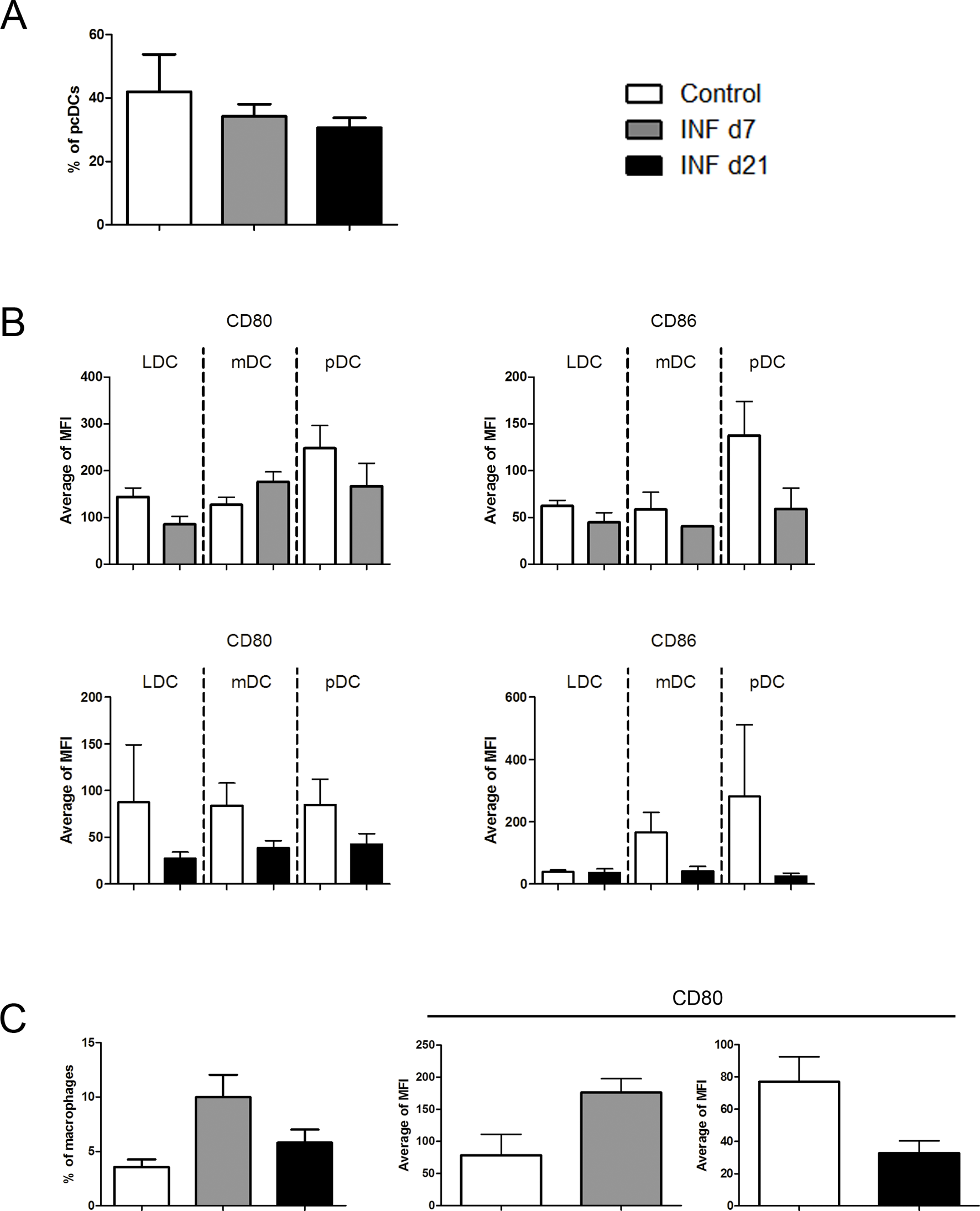

Supplement: Figure S5 — Infection with iRBCs and expression of cell surface molecules on APCs inside the liver. CD11c-YFP mice were intraperitoneally infected or not with Pc-iRBCs All animals were divided into three groups: Control (non-infected; open columns); infected, on day 7 of infection (INF d7; grey columns); or infected, on day 21 of infection (INF d21; black columns). Liver cells were stained with MAbs against a panel of surface molecules to identify different types and subtypes of leukocytes as well as the expression levels of some proteins. (A) Percentages of plasmacytoid DCs (pDCs). (B) CD80 and CD86 expression levels in different subtypes of DCs. (C) Percentage of macrophages and CD80 expression levels. These results are representative of 3 repetitions. (TIF) [file pone.0081409.s005.tif]

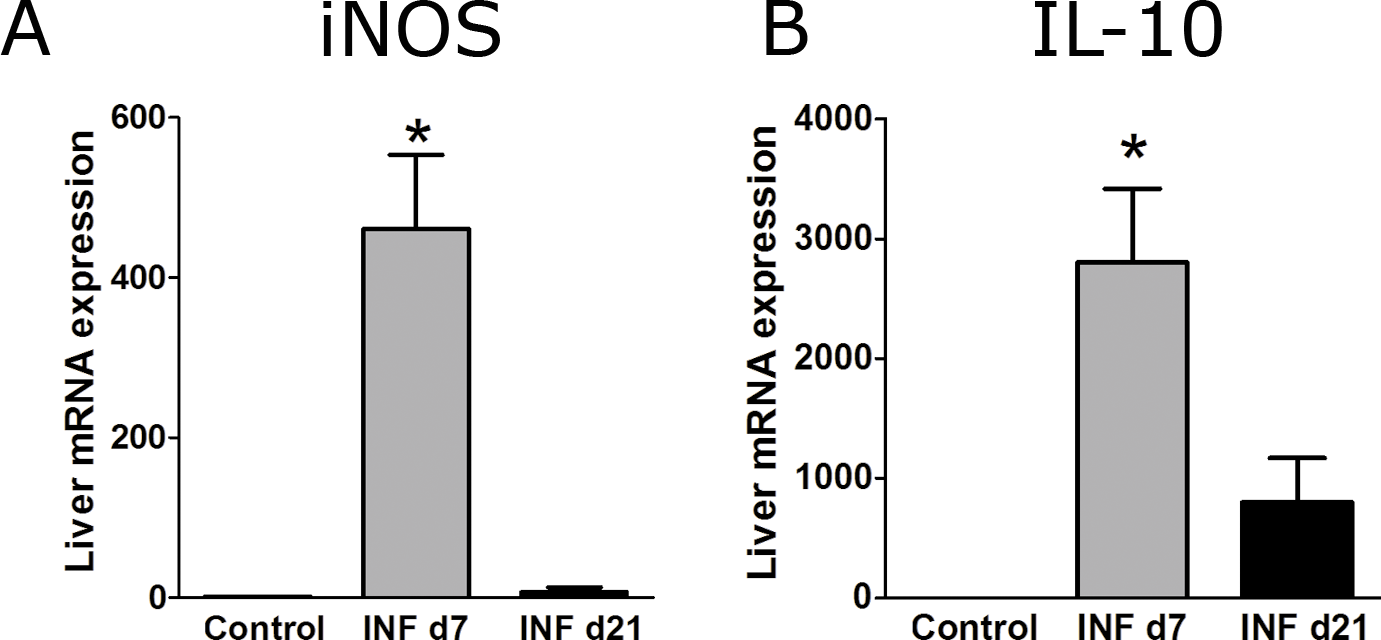

Supplement: Figure S6 — Infection with iRBCs leads to the increased production of mRNA to iNOS and IL-10 inside the liver. Foxp3-GFP mice were intraperitoneally infected or not with Pc-iRBCs. All animals were divided into three groups: Control (non-infected; open columns); infected, on day 7 of infection (INF d7; grey columns); or infected, on day 21 of infection (INF d21; black columns). Liver samples were frozen to further RT-PCR experiments. (A) iNOS mRNA levels. (B) IL-10 mRNA levels. These results are representative of three independent experiments. (TIF) [file pone.0081409.s006.tif]
